# Oxidative Stress and Phototherapy in Atopic Dermatitis: Mechanisms, Role, and Future Perspectives

**DOI:** 10.3390/biom12121904

**Published:** 2022-12-19

**Authors:** Francesco Borgia, Federica Li Pomi, Mario Vaccaro, Clara Alessandrello, Vincenzo Papa, Sebastiano Gangemi

**Affiliations:** 1 Department of Clinical and Experimental Medicine, Section of Dermatology, University of Messina, 98125 Messina, Italy; 2 Department of Clinical and Experimental Medicine, School and Operative Unit of Allergy and Clinical Immunology, University of Messina, 98125 Messina, Italy

**Keywords:** atopic dermatitis, oxidative stress, phototherapy, reactive oxygen species, ultraviolet, UVB, UVA, PON1, NRF2, skin disease

## Abstract

Atopic dermatitis is a chronic inflammatory skin disease in which the overproduction of reactive oxygen species plays a pivotal role in the pathogenesis and persistence of inflammatory lesions. Phototherapy represents one of the most used therapeutic options, with benefits in the clinical picture. Studies have demonstrated the immunomodulatory effect of phototherapy and its role in reducing molecule hallmarks of oxidative stress. In this review, we report the data present in literature dealing with the main signaling molecular pathways involved in oxidative stress after phototherapy to target atopic dermatitis-affected cells. Since oxidative stress plays a pivotal role in the pathogenesis of atopic dermatitis and its flare-up, new research lines could be opened to study new drugs that act on this mechanism, perhaps in concert with phototherapy.

## 1. Introduction

### 1.1. Atopic Dermatitis Pathogenesis: Role of Oxidative Stress

Atopic dermatitis (AD) or atopic eczema is a relapsing–remitting inflammatory skin condition that frequently occurs in children and has an immune-mediated etiopathogenesis [1]. Skin dryness and intense pruritus are the predominant symptoms. It affects individuals of all age groups, although it often occurs from birth or in the first years of life [2]. AD has a negative impact on quality of life (QoL) since it can cause pain, sleep disturbances, and social and personal relationship impairment, with pruritus being the symptom that most affects patients’ daily activities [3,4]. AD is characterized by immune activation, marked epidermal hyperplasia, and defective barrier function, reflecting underlying alterations in keratinocyte differentiation [5]. AD may be associated with high serum levels of total and specific immunoglobulin E (IgE) against a given allergen (extrinsic form) or with normal–low serum levels of IgE (intrinsic form) [6]. Genetic, immunological, and environmental factors contribute to its pathogenesis [7]. A plethora of central genetic mutations has been demonstrated in the pathogenesis of AD: mutations in structural epidermal barrier proteins, mutations in functional proteins that maintain the epidermal barrier, and mutations in factors that regulate the immune system. Skin barrier alterations, with dysfunction of barrier-related proteins such as filaggrin (FLG), loricrin (LOR), and involucrin (IVL), are the first steps that explain the subsequent sensitization against allergens and the so-called “atopic march” in the “extrinsic” form of AD. Alteration of the skin barrier is also related to alteration of the intercorneocyte lipid composition, with further transepidermal water loss. This contributes to the skin dryness typical of AD patients and increased penetration of allergens and pathogens [8]. This microenvironment contributes, especially in the acute phase, to the activation of the immune system with a predominantly Th2-mediated reaction and the release of proinflammatory cytokines such as tumor necrosis factor (TNF) and interleukins (IL-4, IL-9, IL-22) [8]. Epithelium-derived cytokines, such as thymic stromal lymphopoietin (TSLP), IL-25 and IL-33 also act as alarmins after appropriate stimulation, including oxidative stress (OS), and activate a Th2-mediated response in AD, contributing to the inflammatory state of the skin [9]. The persistence of Th2 inflammation and skin barrier disruption contributes to chronic inflammation and to the overproduction of reactive oxygen species (ROS), such as superoxide and hydrogen peroxide. In addition to this mechanism, the increase in ROS may also depend on other exogenous factors such as solar radiation, pollution, psychological stress, and infections [8]. Staphylococcus aureus, a pathogen that frequently causes skin infections and flares in patients with AD because of the disruption the of skin barrier, above all filaggrin deficiency, can sustain skin inflammation through ROS released by monocytes activated by the pathogen itself [8]. Over time, the accumulation of ROS can eventually cause OS, namely an imbalance between the generation of ROS and the mechanisms of the defense of the antioxidant system (AOS). OS may be an intrinsic mediator of amplification and chronicity in AD, as well as in other cutaneous and non-cutaneous diseases including psoriasis, asthma, cystic fibrosis, and cancer [10,11,12,13]. On this topic, chronic inflammatory skin diseases, such as AD or psoriasis, have been related to higher levels of OS markers during flare-ups and/or decreased antioxidant levels [14]. Studies on animals have demonstrated that OS negatively impacts dermal and epidermal microenvironments at different levels. In epidermal keratinocytes, lipid oxidation directly damages DNA, cellular enzymes, or cell membranes, whereas protein and lipid oxidation in the stratum corneum result in skin barrier dysfunction and AD exacerbation. Oxidative direct damage is compounded by the activation of immunological mechanisms, such as the dermal expression of proinflammatory cytokines including IL-6, IL-8, IL-9, and IL-33, and the activation of nuclear factor kappa-B (NF-*κ*B) pathways, which alters skin immune homeostasis and triggers skin inflammation. Finally, a role is played by the pruritogenic stimulation, maybe via activation of transient receptor potential subtype A1 (TRPA1) channels on primary sensory neurons and neurons in dorsal root ganglia with activation of phosphorylation of extracellular signal-regulated kinase in the spinal cord [15].

### 1.2. Marker of Oxidative Stress in Atopic Dermatitis

OS markers identified so far that are involved in AD are aryl hydrocarbon receptor (AHR)/AHR-nuclear translocator (ARNT) system, nuclear factor-erythroid 2-related factor-2 transcription factor (NRF2), myeloperoxidase (MPO) level/paraoxonase (PON)-1 activity [11].

#### 1.2.1. AHR/ARNT-NRF2 Crosstalk

AHR/ARNT system is expressed in the skin. After binding several exogenous ligands, AHR migrates into the nucleus and binds ARNT, thus leading to changes in gene transcription. AHR/ARNT system fortifies the skin barrier by upregulating filaggrin expression. Different AHR ligands can activate other nuclear pathways, with crosstalk between AHR and the antioxidative nuclear factor E2-related factor 2 (NRF2) [16]. IL-4 and IL-13, cytokines of the Th2-mediated response, inhibit the expression of skin adhesion molecules through the phosphorylation of signal transducer and activator of transcription (STAT)-6. This signaling pathway reduces, along with C-C motif chemokine ligand (CCL)-17 and CCL22, the AHR-mediated transcription of FLG, LOR, and IVL [17]. The activation of STAT6 also amplifies the recruitment of Th2 cells in AD skin lesions [18]. Evidence suggests the role of some topical psoriasis treatments, such as coal tar, in blocking the expression of STAT6 via the NRF2 signaling pathway, acting as ligands of AHR [19]. Besides, the link between coal tar and AHR contributes to modifying the skin microbiome composition, which has an important role in the pathogenesis of inflammatory skin diseases [19]. NRF2 also acts on activated macrophages reducing the activity of IL-1β and IL-6, resulting in an anti-inflammatory effect [20]. Therefore, AHR appears to play a pivotal role in regulating the pathogenetic mechanisms of AD, being associated with significant interference with Th2 cytokines, IL-4, and IL-13. Furthermore, the stimulation of AHR by agonists has been found to promote immune tolerance through the differentiation of T-reg cells [19]. In fact, single nucleotide polymorphisms (SNPs) of the AHR gene, modifying the activity of this receptor and its antioxidant capacity, correlate with an increased risk of the onset of AD. No relationship between NRF2 SNPs with AD pathogenesis has been found [21]. However, the AHR/ARNT axis appears to be involved in the development of pruritus in AD since the ARNT gene encodes the neurotrophic factor artemin responsible for epidermal hyperinnervation and pruritus [18].

#### 1.2.2. PONs

Lipid oxidation and damage of the keratinocytes and stratum corneum are involved in the processes of barrier disruption of the skin in AD. PON is a group of enzymes with paraoxonase activity, which act as antioxidants and consequently have anti-inflammatory effects in various diseases such as atherosclerosis and cardiovascular diseases. They hydrolyze lipid peroxidation products generated during OS. PON1, 2, and 3 have been identified and they are involved in a plethora of diseases, such as PON3 in the process of atherosclerosis [22]. Compared to PON1 and PON3, PON2 contributes to innate immunity by destroying bacterial signaling molecules that promote bacterial proliferation [23]. PON1 activity takes place within the high-density lipoprotein (HDL)/apoliprotein A1(ApoA1)/PON1 complex in regulating immune responses. ApoA1 regulates the balance between Th17 and Tregs and improves mitochondrial functions. Lipid peroxidation of HDL is the expression of the dysregulation of the OS mechanisms, and it is carried out by the enzyme MPO, released in circulation by activated leukocytes. Alteration of anti-oxidative mechanisms in AD patients is supported by the finding of high levels of MPO, low levels of circulating PON1, and significantly increased MPO/PON1 ratio [24]. These findings suggest that patients with chronic AD have an altered lipid profile and reduced PON1 levels [25] which protect the immune cell membrane from lipid peroxidation and mitochondria from circulating oxidized lipoproteins and oxidative damage [20].

**Figure 1:**
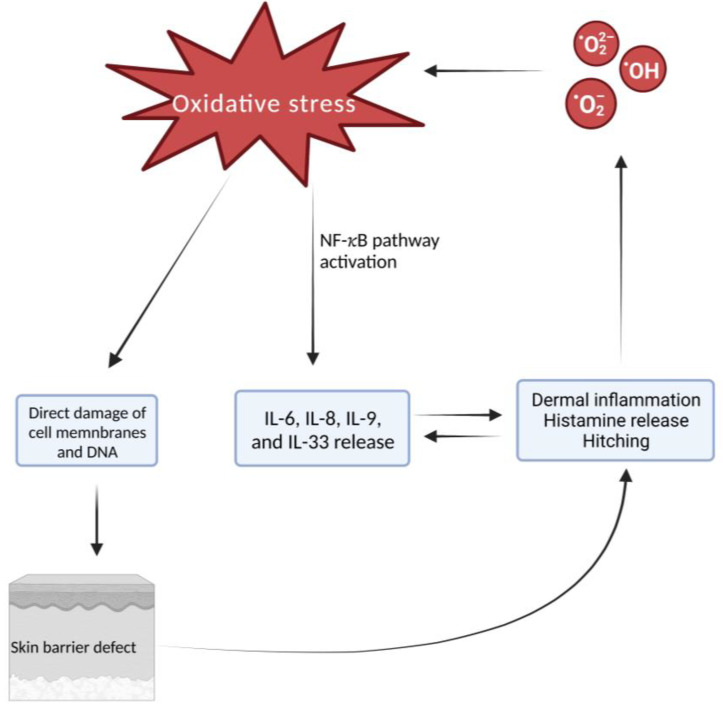
Oxidative stress causes direct damage to the cell membrane and DNA which leads to skin barrier defect. Furthermore, the activation of the NF-*κ*B pathway leads to the release of IL-6, IL-8, IL-9, and IL-33. The defect of the skin barrier causes inflammation of the dermis and release of histamine and itching, leading in turn to an increased release of IL-6, IL-8, IL-9, and IL-33. All these mechanisms determine a further increase in the release of ROS, establishing a vicious circle that is self-maintaining. Created with BioRender.com.

Dermal inflammation is the hallmark of AD in affected areas, which could be enhanced by OS. OS can activate nuclear factor kappa- B (NF-*κ*B) pathways to induce gene expression and synthesis of antioxidant enzymes. NF-*κ*B pathway activation also induces the expression of proinflammatory cytokines, such as IL-6, IL-8, IL-9, and IL-33, which in turn enhances dermal inflammatory infiltrate and histamine release in the affected skin, thus worsening symptoms. OS can directly damage epidermal keratinocytes through DNA damage, damage to cellular enzymes, or damage to cell membrane structures through lipid oxidation. Epidermal edema or spongiosis and disrupted stratum corneum represent the consequence of these intracellular changes [25]. Figure 1 represents the mechanisms through which OS acts in AD.

## 2. Atopic Dermatitis Treatment

### 2.1. Topical, Systemic, and Biological Treatment

First-line therapy for the acute management of AD includes topical corticosteroids (TCS) whose long-term use is however limited by a plethora of possible side effects, including localized skin atrophy, telangiectasias, perioral dermatitis, and iatrogenic acne [26]. Hence, it derives non-steroidal alternatives, among them topical calcineurin inhibitors (TCIs), such as tacrolimus and pimecrolimus, which are approved for short-term or intermittent administration in patients who have previously failed on, or have contraindications to, TCSs [27,28]. TCIs lead to the inhibition of T cell activation and to the downregulation of pro-inflammatory cytokines, with a consequent immunosuppressive action [27]. TCIs can cause local adverse effects such as skin burning and irritation, even if they do not cause the risk of local atrophy [26]. Among the non-steroidal alternatives, phosphodiesterase 4 (PDE4) inhibitors are being largely used. They increase the levels of cyclic adenosine monophosphate in AD skin thus reducing the expression of proinflammatory cytokines. Crisaborole 2% ointment has been demonstrated to be safe and effective [29,30], whereas roflumilast cream, another anti-PDE4 inhibitor, already approved for psoriasis therapy, is under clinical investigation for AD treatment [31]. In addition, difamilast ointment completed phase III trials in both the adult and pediatric populations, with a statistically significant improvement in AD-related lesions compared to the control group [32]. As already said, cytokines implicated in AD pathogenesis, such as IL-4, IL-13, and IL-31, signal through the intracellular janus kinase (JAK)-STAT pathway and share activation of JAK1, suggesting the value of JAK inhibitors (JAKi) as a promising therapeutical approach [33]. Among them, the topical cream of ruxolitinib, a JAK1/JAK2 inhibitor, represents a new promising topical tool for short-term chronic treatment for patients with mild to moderate AD [34]. Delgocitinib ointment, a pan-JAK inhibitor, is the world’s first approved topical JAKi for the treatment of AD both in adults and children [35]. Finally, tapinarof cream 1%, an AHR agonist, is being studied for the treatment of AD, showing a significant improvement in the signs and symptoms of the disease [36,37,38]. Switching to oral therapies, oral corticosteroids are effective options but can only be used for a few weeks due to their long-term side effects. The utility of oral cyclosporine and azathioprine has been well-documented in children and adults with moderate to severe AD refractory to topical therapy [27]. In recent years, medicine has been increasingly developing toward target therapy, with the progressive use of biological drugs that selectively block the cytokines and inflammatory pathways responsible for the disease. Dupilumab, the first biological drug approved by the FDA for moderate-to-severe AD, binds to the IL-4Ra, inhibiting IL-4 and IL-13 signaling, and has demonstrated an improvement of at least 75% on the Eczema Area and Severity Index (EASI), on pruritus, and QoL as compared to placebo [39]. It is now known that IL-13 expression is much higher and more frequently detected in AD skin lesions than IL-4, suggesting that IL-13 could also be an excellent biological target [40]. On this topic, lebrikizumab and tralokinumab, two monoclonal antibodies (mAb) antagonizing IL-13, have demonstrated encouraging clinical efficacy against moderate to severe AD with an excellent safety profile, although they presented a higher risk of conjunctivitis than placebo [41]. In addition to the widely used mAb, other biologics are currently being tested, including an antibody that selectively targets and inhibits IL-31, named nemolizumab [42]. IL-31 is a proinflammatory cytokine that plays a crucial role in mediating pruritus through overexpression of its receptors on sensory nerves [43]. In two phase 3 trials, nemolizumab has achieved an improvement in pruritus and signs of AD for up to 68 weeks [44]. The last FDA-approved treatments are upadacitinib, baricitinib, and abrocitinib, three oral JAKi which show impressive efficacy [42]. They all met primary and secondary endpoints in several studies for moderate to severe AD, proving themselves as promising drugs in the next generation of targeted therapy. Their exceptional effectiveness and speed of action, evaluated with the reduction in EASI, are balanced by a favorable safety profile in clinical studies, with adverse effects reported from mild to moderate; however, data on real-life experience are needed to highlight long-term security, duration, and efficacy [35].

### 2.2. Phototherapy

#### 2.2.1. Ultraviolet Sources

When first-line treatments are unsatisfactory, phototherapy can serve as an efficient option for the management of AD [2]. Phototherapy, classified as “Strength of Recommendation B” and “Level of Evidence II” for the treatment of AD, should be reserved for patients with acute and chronic DA, where behavioral measures, TCS, and TCIs have not yielded clinical benefits. Monotherapy or combination therapy with topical or systemic agents represent the two possible uses. [6,45]. However, numerous factors can limit phototherapy’s usefulness and effectiveness, especially because it requires cycles of bi-weekly sessions, so it can be difficult for patients who live far from centers equipped with this technology [46]. Ultraviolet irradiation can be classified as ultraviolet A (UVA) with the longest wavelengths between 320 and 400 nm, followed by ultraviolet B (UVB) (290–320 nm), and ultraviolet C (UVC) (200–290 nm). UVA is divided into ultraviolet A1 (UVA1, 340–400 nm) and ultraviolet A2 (UVA2, 320–340 nm). The UVB phototherapy is further divided into broadband UVB (BB-UVB, 90–320 nm) and narrowband UVB (NB-UVB, 311–313 nm) [2]. Other forms of phototherapy for AD include psoralen ultraviolet A (PUVA) therapy and UVA1 cold light therapy. PUVA is a photochemotherapy, which consists of UVA radiation with either oral administration of psoralens or topical administration [2]. Compared to other UV phototherapies, NB-UVB has been shown to be more clinically tolerable with fewer side effects compared to other UV phototherapies. NB-UVB causes reduced expression of pro-inflammatory cytokines, downregulation in antigen presentation through inhibition of Langerhans cell activity, and consequent suppression of the lymphocytes T-mediated skin immune system [45]. In addition to AD, this therapy is also indicated for the treatment of psoriasis, parapsoriasis, mycosis fungoides, renal and hepatic pruritus, vitiligo, acute and chronic graft versus host disease, and other skin diseases [46].

#### 2.2.2. Phototherapy Immunomodulatory Effects

As already mentioned, AD is characterized by immune and barrier abnormalities on which NB-UVB exerts its positive effects, thus suppressing the Th2, Th22, and Th1 immune pathways, and through the normalization of epidermal hyperplasia and differentiation, with the consequent elimination of inflammatory leukocytes and Th2/Th22-associated cytokines and chemokines and expression of barrier proteins [47]. Furthermore, phototherapy causes downregulation of cytokines, such as IL-5, IL-13, and IL-31, supporting the hypothesis that these molecules play a crucial role in the pathogenesis of AD and, therefore, may represent possible targets for phototherapy [43]. Moreover, phototherapy induces T-cell apoptosis and dendritic cell reduction [6]. In fact, the T-cell response in the skin of AD patients is predominantly Th2/Th22 even if patients with chronic disease develop a sizable pool of pathogenic Th1. In AD, IL-4 drives IgE secretion mediates recruitment of eosinophils, and attenuates filaggrin expression, whereas IL-22 inhibits keratinocyte maturation. UVB therapy has been demonstrated to improve barrier function by increasing the expression of filaggrin, involucrin, and AMP. Initially, patients experience strong suppression of the Th2/Th22 axis after NB-UVB exposure and, controversially, a decrease in intralesional IL-10 expression [48]. On this topic, IL-10 showed upregulated mRNA expression in both lesional AD and non-lesional AD skin compared with healthy skin, which decreased with NB-UVB. Although IL-10 is implicated in the anti-inflammatory response, its role in AD has been interpreted as part of the predominant Th2 microenvironment, increased levels of IL-10 have been postulated to indirectly contribute to the AMP deficiency in patients with AD, potentially accounting for an increased propensity for infections. Higher IL-10 expression levels were reported in the skin of patients with chronic AD compared with the acute stage, possibly because of an upregulation of the receptors by interferon-gamma (IFN-γ) [49,50].Clinically, the effects of phototherapy have been further demonstrated by the fact that 70% of AD patients received significantly fewer TCS during the 12-month window after finishing NB-UVB, compared to the 12-month window before starting [51]. Phototherapy with medium-dose UVA1 irradiation exerts a significant antipruritic effect, decreases the severity of the disease, and improves the QoL of AD-affected patients. From this, it follows that this technique can be used as a safe and effective treatment [52].

#### 2.2.3. Role of Phototherapy during COVID-19 Pandemic

In the last triennial, coinciding with the pandemic period experienced, the role of phototherapy has gained even more importance because of its therapeutic polyhedricity [53]. Assuming strict adherence to revised procedures for the effective and safe use of phototherapy along with new approaches that improve patient compliance in this historical era, probably the most interesting aspect investigated concerns its antiviral activity [54]. One of the earliest reviews on the subject by Hanna et al. pointed out that, although the antiviral efficacy of ultraviolet blood irradiation (UBI) remained controversial due to the reduced penetrating ability of radiation, indirect confirmation of its potential comes from the effectiveness of Amotosalen/UVA light in minimizing the risk of transfusion-related MERS-CoV transmission through its ability to completely inactivate MERS-CoV in human platelet concentrates [55]. In subsequent work, the same research group confirmed the effectiveness of UV light in reducing MERS-CoV titer below the detection limit in human platelets [56]. Further confirmation of the antiviral potential of photochemotherapy comes from the finding of photoinactivation potential in plasma with Amotosalen and 3 J/cm^2^ of UVA light of six RNA-enveloped viruses, including SARS CoV [57]. Beyond the newly explored therapeutic potential, the promising role of phototherapy is also related to its choice as a viable therapeutic alternative to immunomodulating/immunosuppressive drugs used in AD and similar conditions whose use should be limited because of their ability to impact vaccine-induced immune responses [58,59,60,61] and for their suppressive action on intrinsic antiviral immunity [62,63,64] except for dupilumab, whose evidence of use in the pandemic era is reassuring [65,66,67].

## 3. Atopic Dermatitis and Other Chronic Inflammatory Cutaneous Diseases

In addition to AD, OS role has been demonstrated in several chronic cutaneous diseases, including psoriasis, vitiligo, alopecia areata (AA), lichen planus, pemphigus vulgaris, and skin cancers [68,69,70,71,72]. On this topic, psoriasis is a chronic, immune-mediated inflammatory cutaneous disease, mainly characterized by the presence of erythematous plaques, covered by white scales, especially localized over the extensor zones [73]. Psoriasis shares many features with AD, including immune activation and epidermal hyperplasia. However, major differences in immune polarization exist between these diseases. Although psoriasis is considered a Th1/Th17 disease, AD is predominantly a Th2/Th22-polarized disease with some component of Th1 polarization in the chronic phase and a relative impairment of the Th17 pathway [5]. However, as in DA, the involvement of OS-related molecules and long-lasting inflammation in the induction of keratinocyte proliferation and differentiation has emerged. Being directly exposed to environmental factors, the skin is a major source of free radicals that play a vital role in defending against microorganisms, when at low concentrations. However, when free radical levels increase, they seem to play a role in DNA alteration, cell protein degradation, lipid oxidation, cell apoptosis, tissue damage, impaired T-helper cells response, and subsequent IL-17 secretion, as all these stages are essential in the induction and persistence of psoriasis [74]. Switching to AA, the antioxidant/oxidant balance perturbation represents a trigger mechanism in its pathogenesis, together with emotional and environmental stress. AA is characterized by circumscribed non-scarring hair loss patches, mainly localized in the scalp. Even if its pathogenesis is not totally clear, it is now considered a chronic autoimmune disorder with autoaggressive T cells directed against the anagen hair follicles at the histological examination. Lipid peroxides, a hallmark of OS, and their breaking-down products such as malondialdehyde (MDA) can affect normal cells whose levels strongly correlate with lipid peroxidation levels [75]. Higher thiobarbituric acid-reactive substances (TBARS) levels in plasma, erythrocytes, and scalp biopsies were found in AA patients compared to controls [68,75,76]. Moreover, TBARS tissue levels appeared to be higher in the early phase of the disease and correlated to its gravity. MDA levels in serum and tissue were found to be higher in patients with AA compared with control subjects, and strongly correlated with the severity and longevity of the disease [68].

OS also plays a role in vitiligo, a chronic autoimmune skin disease, characterized by milky white patches mainly localized in visible areas [72,77]. In vitiligo, melanocytes show poor antioxidant capacity due to alterations in antioxidant mechanisms, such as AHR and NRF2/ heme oxygenase-1(HO-1) system, which result in high levels of superoxide dismutase and low levels of catalase [78]. AHR polymorphisms might play a role in Treg cell differentiation, IL-10, IL-17, and IL-22 expression, thus contributing to vitiligo pathogenesis. Higher levels of IL-10 in the serum of tacrolimus-treated patients have been correlated with reduced melanocyte degradation and reduced symptoms. AHR-null mice have been shown to exhibit down-expression of IL-10. The relationship between IL-22 and AHR is known, as the activity of IL-22 is dependent on AHR ligation, whereas the relationship between AHR and IL-17 is so far being defined [19]. Additionally, the role of the NRF2/ Kelch-like ECH-associated protein (KEAP1)-HO-1 pathway in counteracting OS in vitiligo is widely recognized, corroborated by the positive influence of NRF2 polymorphisms [79,80]. This pathway results in antioxidant reactions, though vitiligo melanocytes have reduced HO-1 expression and detoxifying enzymes release, due to reduced nuclear translocation of NRF2 [81,82,83]. This is demonstrated by the increased susceptibility of vitiligo melanocytes to the oxidative insult induced by hydrogen peroxide, which triggers autoimmune and apoptotic phenomena, thus leading to the onset and progression of vitiligo [84]. These remarks have contributed to the current interest in identifying NRF2 modulators as a possible therapeutic strategy [85].

## 4. Potential Targets of Oxidative Stress during Phototherapy in Atopic Dermatitis

**Figure 2:**
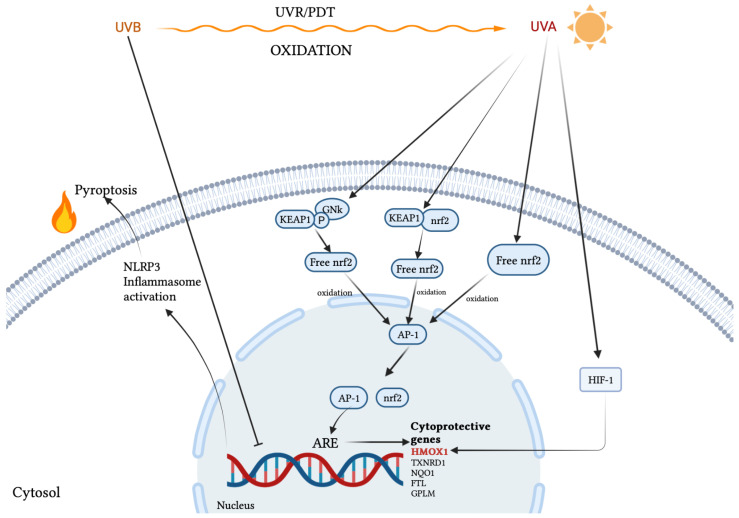
UVR modulation on the expression of cytoprotective genes. The figure shows the diametrically opposite effects of UVA and UVB. UVA-mediated oxidation acts on two different levels by promoting the intracytoplasmic accumulation of free NRF2 which, once oxidized, translocates to the nucleus where, dimerizing with AP-1, it acts as a transcriptional activator of cytoprotective genes. Further upregulation of HMOX-1 is provided by the oxidation of HIF-1. UVB-mediated oxidation indirectly inactivates the transcription of cytoprotective genes through direct DNA damage. The lack of this cytoprotective action in turn results in NLRP3 inflammasome activation and consequent pyroptosis.

A study on the effects of UVA conducted in BALB/c mice identified that sulforaphane is associated with OS and may have a role in treating photoaging through the reduction in matrix metalloproteinase-1 (MMP-1) and activation of NRF2, which controls epidermal inflammation. It is therefore concluded that sulforaphane exerted a therapeutic effect in the AD mouse model by the activation of the NRF2/HO-1 axis. The present study also found that the phosphorylation of JAK2/STAT3 and the expression levels of IL-6, IL-1β, and TNF-α were reduced in the SFN-treated group compared with the AD group [86]. Significant data regarding the relevant influence of this treatment regimen on AHR/ARNT and MPO level/PON1 are not yet available in the literature. Instead, much of the evidence regarding the impact of phototherapy on markers of OS in AD can be attributed to the cytoprotective role of NRF2. Despite their low energetic properties, the biological impact of UVA1 on human skin is not negligible because of their penetration properties and the OS that they are able to induce on a massive scale, thus affecting the skin as a whole [87]. In their recent review, Bernerd et al. highlight the role of NRF2 in partially counteracting UVA1-induced OS through upregulation of NRF2 target genes: heme oxygenase 1 gene (HMOX1), thioredoxin reductase 1 (TXNRD1), NAD(P)H quinone dehydrogenase 1 (NQO1), ferritin light chain (FTL), glutamate-cysteine ligase regulatory subunit (GCLM), aldo-keto reductases 2/3 (AKR1C2 and AKR1C3), showing the validity of such involvement both in vivo and in a three-dimensional reconstructed human skin model [88]. Interestingly, the same defense mechanisms induced by OS in the dermis and completely differentiated epidermis also concern cancer cells, suggesting in this sense a multi-cytotype protective role of the NRF2 pathway in coping with the photoinduced OS. Broekgaarden et al. attribute to at least five interconnected pathways the survival mechanisms of cancer cells following photodynamic therapy (PDT) or similar approaches such as ultraviolet light irradiation. Among these NRF2 seems to be a fundamental trigger in restoring the redox balance, thus promoting a prolonged survival of tumor cells [89]. The mechanism by which this happens would see the direct involvement of PDT in oxidizing the NRF2-binding domain of KEAP1, bound to the cytoplasmic cytoskeleton, promoting the cytoplasmic accumulation of free NRF2 which oxidized, it acquires nuclear translocation capabilities, where, dimerizing with activator protein 1 (AP-1), binds to antioxidant response element (ARE) sequences, then triggering the transcription of genes involved in the synthesis of antioxidant agents and the removal of harmful oxidation products. The dissociation of the NRF2-KEAP1 complex is simultaneously reinforced by additional phosphorylation mediated by Jun N-terminal kinase (JNK)-1, induced by PDT [89]. Among the NRF2/AP-1 target genes, the HMOX1 gene encoding HO-1 is in turn upregulated by hypoxia-inducible factor 1 (HIF-1), another transcriptional factor induced by PDT. HO-1 acts as an antioxidant both directly, neutralizing some species of ROS, and indirectly, promoting the formation of bilirubin from heme [89]. It must, however, be clarified that not all ultraviolet radiations indiscriminately determine NRF2-mediated signaling activation. In contrast to the aforementioned action of UVA, several pieces of evidence would seem to correlate UVB exposure with reduced NRF2 activity and thus with the expression of its target genes in normal human epidermal keratinocytes and melanocytes, as well as in dermal fibroblasts [90]. Such poor activating or even inhibitory power of the NRF2 signaling pathway has been related to the ability of UVB photons in inducing direct DNA damage, consequently inhibiting or delaying the activation of NRF2-driven genes and in parallel promoting, in the absence of such survival mechanisms, OS-induced apoptosis or rather an inflammation-dependent cell death (pyroptosis) elicited by NLR family pyrin domain containing 3 (NLRP3) inflammasome activation in keratinocytes after sensing UVB-induced DNA damage [91]. Table 1 summarizes the main OS markers involved in cytoprotective pathways and damaging cell mechanisms. The main actions of UVR are represented in Figure 2.

## 5. Conclusions and Future Perspectives

This study aims to evaluate the role of OS in AD, linking it with the possible antioxidant mechanisms of phototherapy as a therapeutical choice. The dosage of OS-related molecules could prove useful to recognize the activity and severity of various chronic conditions, such as atopic eczema and psoriasis, and to evaluate, not only clinically, the response to systemic therapies, with the aim of identifying potential interactions among ROS that still need further evaluation. Furthermore, these findings could be useful in developing novel therapeutical approaches which could include using antioxidants, possibly together with already validated drugs, in order to obtain complete clearance of the disease and improve the QoL of affected patients. Since OS plays a pivotal role in the pathogenesis of AD and its flare-up, new lines of research could be opened for the study of new drugs that act on this mechanism, perhaps in concert with phototherapy. Of course, it is imperative to conduct further studies evaluating the correct pathways triggered or inhibited by them, to produce safer results and comprehensive treatments for patients suffering from such burdensome diseases.

## Figures and Tables

**Table 1:** Study characteristics.

Topic	Author, Reference	Os Marker	Cytoprotective Mechanism	Cellular Damage Mechanism
UVA1	Bernerd et al. [31]	NRF2	Upregulation of NRF2 target genes: HMOX1, TXNRD1, NQO1, FTL, GCLM, AKR1C2, AKR1C3	//
PDT	Broekgaarden et al. [32]	NRF2	Activation of JNK and NRF2-KEAP1 dissociation. Activation of ARE sequences by NRF2-AP1 and transcription of NRF2 target genes. Upregulation of HMOX1 HIF-1-mediated genes.	//
UVB	Ryšavá et al. [33]	NRF2	//	Direct DNA damage and downregulation of NRF2 target genes
UVB	Vieyra-Garcia et al. [34]	//	//	NLRP3 inflammasome activation

## Data Availability

Not applicable.
